# Factors Associated With *Cryptosporidium* Infection Among Adult HIV Positive Population in Contact With Livestock in Namwala District, Zambia

**DOI:** 10.3389/fpubh.2020.00074

**Published:** 2020-03-13

**Authors:** Ntazana N. Sinyangwe, Joyce Siwila, John B. Muma, Mumbi Chola, Charles Michelo

**Affiliations:** ^1^School of Public Health, University of Zambia, Lusaka, Zambia; ^2^Department of Clinical Studies, School of Veterinary Medicine, University of Zambia, Lusaka, Zambia; ^3^Department of Disease Control, School of Veterinary Medicine, University of Zambia, Lusaka, Zambia

**Keywords:** *Cryptosporidium*, HIV patients, prevalence, risk factors, Zambia

## Abstract

*Cryptosporidium* spp. is one of the leading causes of diarrhoeal disease globally. In Zambia, the burden of *Cryptosporidium* infection in the general human population is unknown and factors associated with it are unclear. A study was conducted to determine the prevalence of *Cryptosporidium* spp. and identify factors associated with its infection among Human Immunodeficiency Virus (HIV) positive individuals in contact with livestock in Namwala district of Zambia. Three hundred and twenty six stool samples were collected from HIV infected individuals presenting at local health centers in Namwala district of Zambia between August 2015 and June 2016. The Meriflour *Cryptosporidium*/*Giardia* test kit was used to test for presence of oocysts. Demographic information such as age and sex and information on hypothesized risk factors was collected using a structured questionnaire. Overall prevalence of *Cryptosporidium* infection was 9.5% (95% CI = 6.7–13.2%); 13.3% and 7.1% among male and female participants, respectively. Males were 2.5 times more likely to be infected than females whereas the divorced had higher odds of being infected (OR = 14.8). Participants who kept animals had a higher prevalence (11.4%) than those that did not (7.0%). Those that shared water with neighbors were 5.7 times more likely to be infected than those who did not. We conclude that *Cryptosporidium* infection is prevalent among HIV positive adults in Namwala district and infection is associated with sex, marital status and sharing water sources among neighbors. Community sensitization is required to create awareness and reduce human exposure to *Cryptosporidium* infection.

## Introduction

*Cryptosporidium* infection is one of the important causes of diarrhoeal illnesses worldwide. It particularly afflicts young children and immune-compromised patients (1), more so in developing countries (i.e. Africa, Asia, and South America) with high prevalence of HIV/AIDS (2, 3).

Globally, the exact burden of cryptosporidiosis is still uncertain due to inactive surveillance. However, there is some information from different regions worldwide (4). In Zambia and the subregion, the burden of *Cryptosporidium* infection in the general human population is unknown as most studies were focused on specific groups of people such as children, or HIV infected persons (5–7). The factors associated with this infection are equally unclear, however, studies elsewhere have postulated that contact with animals, international travel and concurrent immunosuppressive disease are associated with infection (8). It is also reported that close contact among humans particularly under poor hygienic conditions tend to favor parasite transmission (9, 10). Consequently, failure to correctly diagnose cryptosporidiosis is fatal, especially for immune compromised HIV sero-positive individuals. In the various provinces and districts of Zambia, information on the exact burden is limited because previous studies mainly focused on children below the age of five (11–13), and farm workers (14). In adults, a study done from four townships in Lusaka province reported that 15 of the 16 adult patients diagnosed with cryptosporidiosis were HIV sero-positive (15), indicating a high number of cases in these patients. The prevalence of HIV/AIDS in Zambia is estimated to be 13% (16).

*Cryptosporidium* infection has been reported in animals in Zambia (2, 17–19). In Namwala district, majority of the population are livestock farmers (16). With the increase in livestock rearing in Namwala and reported zoonotic *Cryptosporidium* species in livestock (17, 19), there was need to determine the burden of *Cryptosporidium* infection particularly among immune compromised adult HIV patients in contact with livestock. This study was therefore aimed at determining the prevalence of *Cryptosporidium* spp. and identifying associated risk factors among the adult HIV positive population in contact with livestock in Namwala district of Zambia.

## Materials and Methods

### Study Area

The study was conducted in Namwala district in the Southern province of Zambia, located on the Kafue River. Southern province was selected due to the high cattle population in the area. According to the Central Statistical Office (CSO), the cattle population in Southern province stands at 1,293,715 (20). Namwala is predominantly rural with extensive human-cattle interaction from traditional cattle rearing commonly practiced in the area. Estimated human population of the district is 106,834 (16) while cattle population is estimated to be 300,000 herds (21).

Namwala district has one general hospital and 12 rural health centers (from which four centers were selected for the study). It also has one District Veterinary Office with 12 Veterinary Camps that are manned by veterinary assistants.

### Study Design and Study Population

This was a cross-sectional health facility based survey conducted between August 2015 and June 2016 among consenting adult (≥18 years old) HIV patients. The participants were drawn from HIV patients receiving Anti Retro-viral Treatment (ART) (for more than 6 months) from Namwala District Hospital, Maala Rural Health Centre, Chitongo Rural Health Centre, and Kabulamwanda Rural Health Centre. Eligible participants who lived in Namwala for <6 months, did not consent, were pregnant and persons who were on anti-protozoal treatment were excluded from the study.

### Sampling and Sample Size Estimation

The estimated sample size was 388 using the formula: n = z^2^ × *p* (1−*p*)/ϵ (22), with the assumption that the estimated *Cryptosporidium* infection in the HIV infected adult population in Zambia was 28 (23), with 95% Confidence Interval and 80% power of the study. Sample size was distributed across the health centers using probability proportional to size. ART patient registers served as the sampling frame.

About 10–20 g of stool sample was collected from each of the 326 participants in stool specimen containers. Samples were collected from health facilities and immediately submitted to the research team after labeling. These were preserved in 10% formalin and later transported in cooler boxes to the University of Zambia, School of Veterinary Medicine, Public health laboratory and the Central Veterinary Research Institute (CVRI) for analysis. A pre-tested close-ended questionnaire was used to collect data on demographic parameters (age, sex, education, employment status, and income), water and sanitation and farm management practices likely to increase the risk for *Cryptosporidium* infection.

### Laboratory Sample Analysis

The Meridian Merifluor® *Cryptosporidium/Giardia* kit (Meridian Diagnostics Inc., Cincinnati, Ohio, USA) was used for detection of *Cryptosporidium* oocysts in the fecal samples according to manufacturer's instructions. The diagnostic kit detects *Cryptosporidium* oocysts and *Giardia* cysts and the two were differentiated based of size and shape. The test has a high specificity and sensitivity (24). Slides were examined using an epi-fluorescence microscope (Olympus CX31). A sample was considered positive if at least one oocyst was visualized under the microscope, with the characteristic apple green fluorescence.

### Data Analysis

Questionnaire data and laboratory results were checked for completeness and entered in Microsoft Excel. Data was then exported to STATA® statistical package version 13.0 for coding and analysis. Descriptive statistics were generated for all the variables under study. Chi square and Fisher's exact tests (where applicable) were used to test for associations between the disease outcome and the hypothesized risk factors in univariate analysis. Further analysis was done using multiple logistic regression to determine predictors of *Cryptosporidium* infection. All results were considered significant at *p* ≤ 0.05.

## Results

### Socio-Demographic Factors and Prevalence of *Cryptosporidium* in HIV Positive Patients

Of the 326 participants, 61% (198/326) were females while 39% were males. Majority (51%) of the participants was aged between 30 and 49 years and the mean age was 35.6 ± SD 11.8. About 71% of the participants were married. Most participants had attended primary education (64%) with only three percent being in formal employment. Farming, which included both livestock rearing and crop cultivation, was the major occupation (70%), followed by trading (28%).

The overall prevalence of *Cryptosporidium* infection among adult HIV positive patients was 9.5% (95% CI: 6.7–13.2%). This varied according to sex (*p* = 0.06), being higher among males (13.3%; 14/128) compared to females (7.1%; 17/198). Variation in prevalence was also observed among the different age groups (Table 1) but the difference was not statistically significant (*p* = 0.25). A statistically significant association was observed with marital status (*p* = 0.02) with the divorced group having a higher prevalence compared to other categories. When the prevalence of *Cryptosporidium* was compared in the four sampled areas, there was no difference in the infection rates (*p* = 0.52) (Table 1).

**Table 1:** Association of *Cryptosporidium* infection and socio-demographic characteristics for the adult HIV positive population in Namwala district (*n* = 326).

**Variable**	***n* = 326**	***Cryptosporidium* positive (%)**	***P*-value**
Study area			
Namwala central	101	9 (8.9)	
Chitongo	15	1 (6.7)	0.52
Kabulamwanda	100	13 (13.0)	
Maala	101	8 (7.2)	
Sex			
Female	198	14 (7.1)	0.06
Male	128	17 (13.3)	
Age			
<30	120	8 (6.7)	
30–49	167	17 (10.2)	0.25
50+	39	6 (15.4)	
Marital status			
Single	29	1 (3.5)	
Married	232	18 (7.8)	0.02
Divorced	31	8 (25.8)	
Widow	34	4 (11.8)	
Education			
None	53	6 (11.3)	
Primary	171	15 (7.2)	0.14
Higher than primary	55	10 (15.2)	
Employment			
Yes	9	1 (11.1)	0.60
No	317	30 (9.5)	
Income^*^			
None	317	30 (9.5)	
<500	4	1 (25.0)	0.60
500–1,000	3	0	
1, 001–5, 000	2	0	
Occupation			
Farming	228	24 (10.5)	
Trading	93	7 (7.5)	0.72
Others	5	0	

* *Zambian Kwacha*.

### Environmental Factors and Prevalence of *Cryptosporidium* in HIV Positive Patients

Table 2 summarizes associations of *Cryptosporidium* infection and environmental characteristics considered among adult HIV positive patients. Household water sources, treatment of water before consumption, treatment method and sharing water source with animals were not significantly associated *Cryptosporidium* infection. However, sharing water source with their neighbors was significantly associated with infection (*p* = 0.01).

**Table 2:** Association of *Cryptosporidium* and environmental characteristics for the adult HIV positive population (*n* = 326).

**Variable**	n^*^	***Cryptosporidium* positive (%)**	***P*-value**
Water source			
Borehole			
Yes	238	24 (10.1)	0.56
No	88	7 (8.0)	
Shallow well			
Yes	91	8 (8.8)	0.78
No	235	23 (9.8)	
Stream/river			
Yes	32	2 (6.2)	0.75
No	294	29 (9.9)	
Municipal supply			
Yes	3	0	1.00
No	323	31 (9.6)	
Water treatment method			
Borehole			
Chlorination	25	2 (8.0)	
Boiling	0	0	0.90
None	213	22 (10.3)	
Shallow well			
Chlorination	20	1 (5.0)	
Boiling	2	0	0.91
None	70	7 (10.0)	
Stream/river			
Chlorination	15	1 (6.7)	
Boiling	0	0	1.00
None	17	1 (5.9)	
Municipal supply			
Chlorination	0	0	
Boiling	0	0	1.00
None	3	0	
Water source shared with neighbors			
Yes	317	28 (8.8)	0.01
No	9	3 (33.3)	
Water source shared with animals			
Yes	165	18 (10.9)	0.38
No	161	13 (8.1)	

* *Number sampled*.

### Farm Management Practices and Prevalence of *Cryptosporidium*

There was no statistical difference (*p* = 0.18) in *Cryptosporidium* infection rates between participants who kept animals (11.4%; 21/184) and those that did not (7.0%; 10/142), even though the infection was relatively higher in those that kept animals (11.4%). It was further observed that *Cryptosporidium* infection in participants was similar whether their animals or farm had a diarrhea episode or not for all the different animal species kept (Table 3). Type of husbandry (*p* = 1.00), contact with animals (*p* = 0.23) and the type of contact (*p* = 1.00) for the different animal species kept on the farms had no influence on *Cryptosporidium* infection among the HIV positive individuals. Furthermore, other factors investigated such as contact with livestock by family members (*p* = 0.45) and whether neighbors kept animals (*p* = 0.71) showed no relationship with *Cryptosporidium* infection among the HIV positive individuals (Table 4).

**Table 3:** Association of *Cryptosporidium* infection and husbandry practices among adult HIV positive population of Namwala district, Zambia (*n* = 326).

**Variable**	**Calves**		**Lambs**		**Kids**		**Piglets**		**Chicks**	
	n^*^	**+ve (%)**	n^*^	**+ve (%)**	n^*^	**+ve (%)**	n^*^	**+ve (%)**	n^*^	**+ve (%)**
Contact										
Yes	80	9 (11.2)	1	0	15	1 (6.7)	6	1 (3.2)	38	2 (5.3)
No	53	7 (13.2)	6	1 (16.7)	28	3 (10.7)	10	2 (20.0)	26	4 (26.7)
Type of contact										
Feeding	35	4 (11.4)	1	0	10	0	2	0	23	1 (4.4)
Cleaning	11	1 (9.1)	0	0	0	0	4	1 (25.0)	2	1 (50.0)
Others	34	4 (11.8)	0	0	6	1 (16.7)	0	0	13	0
Diarrhea										
Yes	28	6 (21.4)	0	0	3	0	1	1 (100)	1	0
No	103	9 (8.7)	2	0	35	2 (0.6)	14	1 (7.1)	53	7 (13.2)
Husbandry type										
Intensive	0	0	0	0	0	0	0	0	0	0
Semi-intensive	28	4	0	0	18	1 (5.5)	6	1(16.7)	21	2 (9.5)
Extensive	122	13	8	0	26	2 (7.7)	13	2 (15.4)	37	5 (13.5)

* Number sampled.

*+ve = positive for Cryptosporidium*.

**Table 4:** Relationship between *Cryptosporidium* infection in HIV positive individuals and contact with livestock among family members in Namwala district, Zambia (*n* = 326).

**Variable**	n^*^	**No positive (%)**	***P*-value**
Contact with livestock			
Yes	179	19 (10.6)	0.45
No	147	12 (8.2)	
Type of contact			
Heading/milking	143	16 (11.2)	
Plowing	13	1 (7.7)	0.92
Feeding	19	2 (10.5)	
Veterinary assistance	1	0	
Cleaning animal quarters	3	0	
Neighbors keeping animals			
Yes	302	30 (9.9)	0.71
No	24	1 (3.2)	

* *Number sampled*.

*No, Number*.

### Association Between *Cryptosporidium* Infection and Diarrhea

About 19% of the participants or their family members reported having had a diarrhea episode 3 weeks prior to the study; 6% of these experienced diarrhea after having prior contact with an individual or animal suffering from diarrhea. Prevalence of *Cryptosporidium* in participants with diarrhea was not different from that in participants with no diarrhea (9.8% vs. 9.4%; *p* = 0.92).

### Predisposing Factors of *Cryptosporidium* Infection

Employing multivariate analysis, sex, marital status and sharing water source with neighbors were significantly associated with *Cryptosporidium* infection (Table 5). Males were 2.5 times more likely to be infected with *Cryptosporidium* than females (*p* = 0.02). The divorced were 14 times more likely to be infected than those who were single (*p* = 0.02). Furthermore, sharing water source with neighbors was also a significant predictor of *Cryptosporidium* infection (OR=5.7; *p* = 0.03) (Table 5).

**Table 5:** Factors associated with *Cryptosporidium* infection in the adult HIV population in Namwala District (*n* = 326).

**Character**	**n**	**OR^**a**^ (95% CL)**	***P*-value**
Sex			
Female	198	Ref	
Male	128	2.5 (1.13–5.70)	0.02
Marital status			
Single	29	Ref	
Married	232	3.0 (0.37–24.9)	0.30
Divorced	31	14.8 (1.58–138.4)	0.02
Widowed	34	5.90 (0.57–60.5)	0.13
Water source shared with neighbors			
No	9	Ref	0.03
Yes	317	5.7 (1.15–27.9)	

*OR^a^, adjusted Odds Ratio*.

Table 6 shows the number of infected males and female individuals disaggregated based on age, marital status and contact with animals. There was a significant difference in the number of infected males and females based on age and marital status. The number of infected individuals based on material status and whether they kept animals or not was also significantly different. The number of *Cryptosporidium* positive individuals was higher for those that kept animals (Table 6).

**Table 6:** Cross tabulation of male and female participants infected with *Cryptosporidium* according to age, marital status, and contact with animals in adult HIV population in Namwala District (*n* = 326).

**Variable**		**Sex**		**Contact with animals**	
	**n (no +ve)**	Female^*^ (no +ve)	**Male^+^ (no +ve)**	**Yes^$^ (no +ve)**	**No^#^ (no +ve)**
Age					
<30	120 (8)	84 (4)	36 (4)	59 (3)	61 (5)
30–49	167 (17)	90 (6)	77 (11)	100 (14)	67 (3)
50^+^	39 (6)	24 (4)	15 (2)	25 (4)	14 (2)
		0.02		0.11	
Marital status					
Single	29 (1)	15 (1)	13 (0)	8 (0)	21 (1)
Married	232 (18)	132 (5)	100 (13)	138 (13)	94 (5)
Divorced	31 (8)	22 (4)	9 (4)	18 (6)	13 (2)
Widowed	34 (4)	29 (4)	5 (0)	20 (2)	14 (2)
		0.01		0.01	
Contact with animals					
Yes	184 (21)	114 (9)	70 (12)		
No	142 (10)	84 (5)	58 (5)		
		0.61			

* Number of females sampled.

+ *Number of males sampled*.

$ *Number of participants keeping animals*.

# *Number of participants not keeping animals*.

## Discussion

This study aimed to determine the prevalence of *Cryptosporidium* spp. and identify associated risk factors among adult HIV positive people in contact with livestock in Namwala district of Zambia. It was established that adult HIV positive people are exposed to *Cryptosporidium* infection, with sex, marital status and sharing water source with neighbors being significant predictors of infection.

The prevalence estimated in this study (9.5%) is lower than that reported in Kenya (34%) (25). This could be attributed to use of a more sensitive polymerase chain reaction (PCR) in the Kenyan study, and the study population comprised of untreated HIV positive individuals. Individuals in the current study were on ART, a treatment known to help in reducing susceptibility to opportunistic infections such as those by *Cryptosporidium* spp.; and reducing duration of associated clinical signs (26). Results also revealed that males were more likely to have *Cryptosporidium* infection than females. Differences in social responsibilities that expose males to infection more than females could be responsible for the variations. For example, males are responsible for herding the animals while females mostly perform other household chores; such social responsibilities may cause increased contact with livestock through herding and milking, although this assertion is not supported by findings from the current study. Other studies have equally reported no relationship between gender and *Cryptosporidium* infection (14, 25).

Significant association was observed between marital status and *Cryptosporidium* infection with those divorced being at higher risk than other groups. The risk was shared between males and females (Table 6), that is, the number infected was the same. This disagrees with Wanyiri (25) who found those married to be at risk, although their results were not further disaggregated. This result is not easily discernible but could be attributed to high stress levels in the divorced group giving way to opportunistic infections. In social setups like those in Namwala, divorced women become vulnerable to material poverty since majority of marriages are customary and most of the financial assets (land and cattle) are usually taken away by the household heads (who are males) after divorce (27). On the other hand, age was not associated with *Cryptosporidium* infection and this supports findings from a previous study in Lusaka, Zambia (2). Individuals aged 50 years and above had a relatively high prevalence than other age groups (Table 1). This could be attributed to the weakened immune system of individuals as they grow older, hence becoming more susceptible to opportunistic infections like *Cryptosporidium* spp. infections (28). Participants who kept animals were twice as likely to be infected as those who did not. Contact with domestic animals, which is expected in people that rear animals especially among small scale farmers (that are unable to outsource labor), could have exposed the participants to *Cryptosporidium* infection. This affirms assertions that domestic animals are possible sources for human infection and contact with these animals is a known risk factor for infection (2, 25, 29). Participants with young animals that had diarrhea 3 weeks prior to the study had a high chance of having *Cryptosporidium* infection. Contact with cattle, especially calves, has been shown to increase the risk of exposure to *Cryptosporidium* (2, 25, 30). Taking into consideration the study area with its high livestock numbers and the current evidence of *Cryptosporidium* infection in the immune-compromised individuals (HIV infected), it is evident that health care providers cannot ignore the fact that routine screening for opportunistic infections in high risk groups should be a priority. Activity surveillance for *Cryptosporidium* infection accompanied by targeted health education will help reduce infections.

Prevalence of *Cryptosporidium* spp. in participants with history of diarrhea in the 3 months prior to the study was marginally higher than those with no diarrhea. Most participants were asymptomatic during sample collection but were probably still shedding oocysts. The finding of slightly high prevalence in diarrhoeic participants concurs with findings from Kenya among HIV positive individuals not yet on ART (25), and Zambian children (31).

Sharing water sources with neighbors was associated with *Cryptosporidium* infection. Inadequate or improper hygiene practices can contaminate water (32, 33), however, the finding could have been by chance as the water sources could have been contaminated along the conveyance lines. It is important to note that recent publications indicate that anthroponotic transmission of *Cryptosporidium* appear to predominate in countries with poor sanitation (10) which could explain the lack of association between infection and sharing of water sources with animals in the current study. Household water source and sharing of water source with animals were not associated with *Cryptosporidium* infection. Most of the household water was sourced from boreholes which have reportedly low oocyst contamination levels (14, 34).

The current findings indicate that *Cryptosporidium* spp. infections are prevalent among adult HIV positive populations in contact with livestock in Namwala district. Infections are driven by socio-demographic factors (sex and marital status) and livestock production activities. The study has documented risk factors that are associated with *Cryptosporidium* infection in HIV infected adults, however, it is important to note that this was a health facility-based study targeting only those that attended ART clinic and therefore, the findings cannot be generalized to the general population. Based on the current findings, we recommend community sensitization on zoonotic aspects of *Cryptosporidium* infection to create awareness and reduce human exposure to infection in the district. Furthermore, health facilities should include screening for opportunistic infections like *Cryptosporidium* during routine ART clinics. Future studies should focus on establishing the species and subtypes circulating among the human and animal populations, information that is necessary to understand the molecular epidemiology of the parasite.

## Data Availability Statement

All datasets for this study are included in the article.

## Ethics Statement

This study was carried out in accordance with the recommendations of Excellence in Research Ethics and Science (ERES) Ref. No. 2015-June-012 with written informed consent from all subjects. All subjects gave written informed consent in accordance with the Declaration of Helsinki. The protocol was approved by the Excellence in Research Ethics and Science (ERES).

## Author Contributions

NS, JS, JM, and CM contributed to the conception and design of the study. NS collected and analyzed the data and drafted the manuscript. NS, JS, JM, and MC participated in the analysis and interpretation of data. NS, JS, JM, CM, and MC were all involved in the preparation of subsequent manuscript drafts and read and approved the final manuscript. NS is guarantor of the paper.

### Conflict of Interest

The authors declare that the research was conducted in the absence of any commercial or financial relationships that could be construed as a potential conflict of interest.
